# Trypsin- and Chymotrypsin-Like Serine Proteases in *Schistosoma mansoni* – ‘The Undiscovered Country’

**DOI:** 10.1371/journal.pntd.0002766

**Published:** 2014-03-27

**Authors:** Martin Horn, Pavla Fajtová, Liliana Rojo Arreola, Lenka Ulrychová, Pavla Bartošová-Sojková, Zdeněk Franta, Anna V. Protasio, David Opavský, Jiří Vondrášek, James H. McKerrow, Michael Mareš, Conor R. Caffrey, Jan Dvořák

**Affiliations:** 1 Institute of Organic Chemistry and Biochemistry, Academy of Sciences of the Czech Republic, Prague, Czech Republic; 2 Center for Discovery and Innovation in Parasitic Diseases, Department of Pathology, University of California San Francisco, San Francisco, California, United States of America; 3 Institute of Molecular Genetics, Academy of Sciences of the Czech Republic, Prague, Czech Republic; 4 Department of Parasitology, Faculty of Science, Charles University in Prague, Prague, Czech Republic; Institute of Parasitology, Biology Centre, Academy of Sciences of the Czech Republic, Ceske Budejovice, Czech Republic; 5 Fraunhofer Institute for Molecular Biology and Applied Ecology IME, Project Group Bioresources, Gießen, Germany; 6 Wellcome Trust Sanger Institute, Wellcome Trust Genome Campus, Hinxton, United Kingdom; University of Melbourne, Australia

## Abstract

**Background:**

Blood flukes (*Schistosoma* spp.) are parasites that can survive for years or decades in the vasculature of permissive mammalian hosts, including humans. Proteolytic enzymes (proteases) are crucial for successful parasitism, including aspects of invasion, maturation and reproduction. Most attention has focused on the ‘cercarial elastase’ serine proteases that facilitate skin invasion by infective schistosome larvae, and the cysteine and aspartic proteases that worms use to digest the blood meal. Apart from the cercarial elastases, information regarding other *S. mansoni* serine proteases (SmSPs) is limited. To address this, we investigated SmSPs using genomic, transcriptomic, phylogenetic and functional proteomic approaches.

**Methodology/Principal Findings:**

Genes encoding five distinct SmSPs, termed SmSP1 - SmSP5, some of which comprise disparate protein domains, were retrieved from the *S. mansoni* genome database and annotated. Reverse transcription quantitative PCR (RT- qPCR) in various schistosome developmental stages indicated complex expression patterns for SmSPs, including their constituent protein domains. SmSP2 stood apart as being massively expressed in schistosomula and adult stages. Phylogenetic analysis segregated SmSPs into diverse clusters of family S1 proteases. SmSP1 to SmSP4 are trypsin-like proteases, whereas SmSP5 is chymotrypsin-like. In agreement, trypsin-like activities were shown to predominate in eggs, schistosomula and adults using peptidyl fluorogenic substrates. SmSP5 is particularly novel in the phylogenetics of family S1 schistosome proteases, as it is part of a cluster of sequences that fill a gap between the highly divergent cercarial elastases and other family S1 proteases.

**Conclusions/Significance:**

Our series of post-genomics analyses clarifies the complexity of schistosome family S1 serine proteases and highlights their interrelationships, including the cercarial elastases and, not least, the identification of a ‘missing-link’ protease cluster, represented by SmSP5. A framework is now in place to guide the characterization of individual proteases, their stage-specific expression and their contributions to parasitism, in particular, their possible modulation of host physiology.

## Author Summary

Schistosomes are blood flukes that live in the blood system and cause chronic and debilitating infection in hundreds of millions of people. Proteolytic enzymes (proteases) produced by the parasite allow it to survive and reproduce. We focused on understanding the repertoire of trypsin- and chymotrypsin-like *Schistosoma mansoni* serine proteases (SmSPs) using a variety of genomic, bioinformatics, RNA- and protein-based techniques. We identified five SmSPs that are produced at different stages of the parasite's development. Based on bioinformatics and cleavage preferences for small peptide substrates, SmSP1 to SmSP4 are trypsin-like, whereas SmSP5 is chymotrypsin-like. Interestingly, SmSP5 forms part of a ‘missing link’ group of enzymes between the specialized chymotrypsin-like ‘cercarial elastases’ that help the parasite invade human skin and the more typical chymotrypsins and trypsins found in the nature. Our findings form a basis for further exploration of the functions of the individual enzymes, including their possible contributions to influencing host physiology.

## Introduction

Schistosomiasis caused by *Schistosoma* blood flukes is a chronic disease with more than 200 million people infected [1]. Schistosome larvae (cercariae), released into an aquatic environment from snail intermediate hosts, penetrate human skin and subsequently develop into adult worms. Adult worms reside in the host vascular system as male/female pairs, and survive for many years, if not decades [2], producing hundreds of eggs per day. Morbidity arises from the host immune responses to eggs in tissues [3]. Treatment relies on one drug, praziquantel, and no effective vaccine has yet been developed [4]. During its complex life cycle, the parasite survives in various environments by presenting or releasing bioactive molecules that aid survival and modulate host physiology [5], [6]. Disruption of these potential mechanisms by specific drugs/vaccines may provide therapeutic benefits.

Proteolysis is a fundamental physiologic process [7], [8]. Proteases (proteolytic enzymes) are crucial to parasitism, including by schistosomes, in facilitating invasion, nutrient intake, hatching, excystment, immune evasion [9], [10] and modulation of host physiology [10]–[15]. Most schistosome research has focused either on cysteine and aspartic proteases (MEROPS database Clans CA and AA, respectively [8]), which are responsible for digesting the blood meal [16], [17] or on the serine proteases (SPs), known as cercarial elastases (CEs; Clan PA, family S1) that facilitate active penetration of the mammalian host [18]–[20].

Regarding the nomenclature for eukaryotic SPs, whereas members of the S1 or ‘chymotrypsin’ family of SPs share a similar tertiary structure, their substrate cleavage specificities differ [8]. Thus, substrate preferences at the P1 subsite [21] may be divided into trypsin-like (P1 preference for basic residues), chymotrypsin-like (bulky hydrophobic residues) and elastase-like (small aliphatic residues) [7].

Despite their name, which was derived from their ability to cleave insoluble elastin, the *S. mansoni* CEs have a chymotrypsin-like P1 specificity [22] due to preferences for phenylalanine and leucine. In contrast to these well-studied CEs [18]–[20], there are fewer descriptions of ‘non-CE’ Clan PA, family S1 serine proteases in *S. mansoni* (SmSPs) [6], [12]–[15], [23], [24].

Among these, SmSP1 (*S. mansoni* serine protease 1, GenBank AJ011561), has been partially described [13], [14]. The open reading frame (ORF) of SmSP1 comprises two non-proteolytic domains, followed by a C-terminal trypsin protease domain. Expression of the trypsin domain (mRNA and protein) was noted in adult worms with a significant accumulation in the tegument (surface) of males [13]. Another SmSP was identified (under TC16843 code) by microarray analysis with a remarkably elevated expression in post-infective larvae (schistosomula) that had been maintained *in vitro* [23]. Two additional biochemical studies support a function for schistosome SPs in modulating host physiology. Specifically, a protein fraction of *S. mansoni* adult worm extracts was shown to possess kallikrein-like protease activity [12]. The isolated native enzyme, termed sK1, cleaved kallikrein substrates and processed kininogen to bradykinin which induced strong vasodilatation and decreased arterial blood pressure in experimental rats; sK1 was found in higher abundance in males [12]. Both, sK1 and SmSP1, are proposed to regulate host vascular functions [6]. In the second study, SP activity in extracts of *S. mansoni* eggs induced significant fibrinolytic activity and was associated with a 27 kDa protein [15]. This protease activity had a similar cleavage pattern to human plasmin and it was hypothesized that the enzyme blocks the intravascular deposition of fibrin by platelets activated by schistosome eggs [15].

In the present study, we sought to understand the gene repertoire of non-cercarial elastase SmSPs by employing a series of genomic, transcriptomic, proteolytic and phylogenetic approaches. In addition to SmSP1, we identified and re-annotated four distinct SmSPs in the *S. mansoni* GeneDB genome database [25], [26] and term them SmSP2 through SmSP5 according to a previous terminology [13]. The data reveal intriguing expression profiles and phylogenetic relationships that stimulate further study of the individual proteases involved, and their contributions to modulating host physiology.

## Materials and Methods

### Ethics statement

Mice are kept in the animal facility of the Biology Center (Academy of Sciences of the Czech Republic) in Ceske Budejovice and all animal experiments are carried out as approved by the Animal Rights Ethics Committee under protocol no. 068/2010 issued according to the national regulation 246/1992 Sb.

### Schistosome material

A Liberian isolate of *S. mansoni* has been maintained in the laboratory by cycling between CD-1 mice and the freshwater snail, *Biomphalaria glabrata*. Mice were subcutaneously injected with 200 cercariae and sacrificed 6–7 weeks post-infection by intra-peritoneal injection of thiopental (50 mg/kg). Adults, eggs and miracidia were isolated as described previously [27]. Cercariae were obtained from infected snails induced to release the parasite under a light stimulus. Cercariae were chilled on ice, collected and transformed mechanically to schistosomula [27], [28], which were then cultured for five days under a 5% CO_2_ atmosphere at 37°C in Basch Medium 169 [29] containing 5% fetal calf serum and 1% ABAM (antibiotics/antimycotics; Sigma-Aldrich). Daughter sporocyst material was isolated by excision of the hepato-pancreases from two month-infected *B. glabrata* snails. The hepato-pancreases from uninfected snails were used as a negative control when evaluation gene expression.

### Isolation of mRNA and cDNA synthesis

Adult worms, eggs, miracidia, daughter sporocysts, cercariae and schistosomula were re-suspended in 500 µl of Trizol reagent (Life Sciences) and processed [30]. Single-stranded cDNA was synthesized from total RNA by SuperScript II reverse transcriptase (Life Sciences) and an oligo dT_18_ primer, and then stored at −20°C.

### Gene annotation, domain expression evaluation and sequencing

Genes encoding complete SmSPs or their specific domains were retrieved from the *S. mansoni* genome database (*S. mansoni* GeneDB, available at http://www.genedb.org/Homepage/Smansoni) through BLAST searches. Amino acid sequences of vertebrate family S1 SPs were used as queries. Specific PCR primers were employed to amplify each of the sequences retrieved, and the respective amplicons cloned into the TOPO TA 2.1 vector (Life Technologies) for propagation in TOP10 *E. coli* cells. For SmSP4 and SmSP5, full-length sequences were obtained by 5′ and 3′ RACE (Rapid Amplification of cDNA Ends, Life Technologies).

Based on more recent annotations, the original sequence information for SmSP4 and SmSP5 (GenBank XM_002572739 and XM_002574902) were corrected in the *S. mansoni* GeneDB database. All newly described SmSP sequences were deposited in GenBank under the accession numbers listed in Table 1. For genes with multi-domain structures, PCR analysis was performed using domain-specific primers in order to detect possible differential expression.

**Table 1:** List of studied serine proteases and their accession numbers.

Name	SchistoDB	GenBank
SmSP1	Smp_030350	KF535923
SmSP2	Smp_002150	KF510120
SmSP3	Smp_103680	KF510121
SmSP4	Smp_129230	KF510122
SmSP5	Smp_141450	KF939306

### Evaluation of gene expressions by RT-qPCR analysis

Gene expression of the SmSPs was assessed using RT-qPCR. For genes with multi-domain structures (SmSP1 and SmSP3), the expression levels of individual domains were evaluated separately. cDNA for various life stages was generated using the mRNA isolation protocol described above and previously [30]. For mRNA isolation, 3 infected *B. glabrata* hepatopancreases and approximately 20 adult pairs, 500 hundred eggs, cercariae and schistosomula were used. Primers for quantitative PCR analysis were designed using the Primer 3 software (http://frodo.wi.mit.edu/ [31],), in order to amplify 150–250 bp regions of the targeted genes or their domains. Primer efficiency was evaluated by serial dilutions of both the primers and the cDNA template as described [32], [33]. Two to three primer pairs were generated per target from which one primer set with optimal efficiency and generating only a single dissociation peak was used (see Supporting Information Table S1).

Reactions, containing SYBR Green I Mastermix (Eurogentech), were prepared in final volumes of 25 µL in 96-well plates [30]. The amplification profile consisted of an initial hot start (95°C for 10 min), followed by 40 cycles comprising 95°C for 30 s, 55°C for 60 s and 72°C for 60 s, and ended with a single cycle of 95°C for 60 s, 55°C for 30 s and 95°C for 30 s. PCR reactions were performed in duplicate for each cDNA sample. At least one biological replicate, i.e., samples from a different RNA isolation was performed for each gene target. Analysis of the cycle threshold (C_T_) for each target was carried out as described [30] and employed *S. mansoni* cytochrome C oxidase I (SmCOX I, GenBank AF216698, [33]) as the sample normalizing gene transcript [27]. Finally, the resulting transcript values were calculated as a percentage of the expression of the normalizing gene (SmCOX I) which was set as 100%. Transcript levels were expressed as log functions and as a percentage relative to that of SmCOX I in order to compare variable expression patterns. The threshold for significance of expression was set to 0.01% of the expression of SmCOX I.

### Phylogenetic analyses of SmSPs

The amino acid sequences of 96 vertebrate and invertebrate members of the S1 serine protease family were aligned in MAFFT [34] using the E-INS-i method, and gap opening (–op) and extension penalties (–ep) of 5.0 and 0.0, respectively. The non-catalytic domains and N-terminal extensions were excluded from the resulting alignment in BioEdit (v7.0.5.2; [35]). The bacterial trypsin from *Streptomyces griseus* was used as an outgroup. The list of family S1 proteases (SPs sequences) used for the phylogenetic analysis is in the Supplementary Table S2. The Maximum Parsimony analysis was performed in PAUP* (v4.b10; [36]), using a heuristic search with random taxa addition, the ACCTRAN option, and the TBR swapping algorithm. All characters were treated as unordered whereas gaps were treated as missing data. Maximum Likelihood analysis was performed in RAxML under the WAG model [37]. Clade support values were calculated from 1000 bootstrap replicates with random sequence additions for both analyses. All trees were displayed using the TreeView32 program [38].

### Collection of E/S products and soluble protein extracts

Fifty pairs of adult worms, 1 000 eggs or 1 000 schistosomula were washed five times in Basch Medium 169 containing 1% Fungizone (Gibco) and allowed to stand for 1 h at 37°C in 5% CO_2_. Samples were washed 10 times and then incubated in the same Basch Medium overnight (adults and eggs) or for five days (schistosomula) at 37°C in 5% CO_2_. Parasite material was then washed 10 times in M-199 medium (alternative medium for schistosoma cultivation without serum and proteins,Gibco) containing 1% ABAM and incubated in the same medium for 16 h at 37°C in 5% CO_2_. Medium containing E/S products was removed and filtered using an Ultrafree-MC 0.22 µm filter (Millipore). Filtered medium was buffer exchanged into ice-cold 1× PBS (pH 7.4) and concentrated at 4°C to a 2 ml final volume by centrifugation at 4000 *g* using an Amicon 10000 Ultra-15 Centrifugal Filter Unit (Millipore). The total volume of PBS used for buffer exchange was 40 ml. Samples (0.04–0.37 mg protein/ml) were frozen in liquid nitrogen and stored at −80°C.

Soluble protein extracts (1–5 mg protein/ml) from *S. mansoni* adults, eggs and 5 day-old schistosomula were prepared by homogenization in 50 mM Tris-HCl buffer, pH 8.0, containing 1% CHAPS, 1 mM EDTA and 10 µM of the cysteine protease inhibitor, E-64, in an ice bath. The extracts were cleared by centrifugation (16,000 *g*, 10 min, 4°C), filtered with an Ultrafree-MC 0.22 µm and stored at −80°C.

### Proteolytic activity measurement

Proteolytic activities were measured in a kinetic continuous assay using the following peptidyl fluorogenic, 7-amino-4-methylcoumarin (AMC) substrates (Bachem) at a 50 µM final concentration: Z-F-R-AMC (Z, Benzyloxycarbonyl), Bz-F-V-R-AMC (Bz, Benzoyl), Z-G-P-R-AMC, P-F-R-AMC, Boc-L-R-R-AMC (Boc, t-Butyloxycarbonyl), Boc-Q-A-R-AMC, Boc-V-L-K-AMC Suc-A-A-F-AMC (Suc, Succinyl), Suc-A-A-P-F-AMC, Suc-L-Y-AMC, MeOSuc-A-A-P-V-AMC (MeOSuc, 3-Methoxysuccinyl), Z-G-G-L-AMC and Z-V-K-M-AMC. Assays were performed at 37°C in 96-well black microplates in a total volume of 100 µl. Parasite extracts (1–3 µg) or E/S products (0.05–1 µg) were pre-incubated for 10 min in 150 mM Tris-HCl, pH 8.0, containing 10 µM E64, 1 mM EDTA in the presence or absence of 0.5 mM of the serine protease inhibitors, Pefabloc SC and PMSF. E64 was included routinely in extract preparations in order to inhibit Clan CA cysteine protease activity that is present in the life-stages examined [30], [39], [40]. Hydrolysis of substrate was measured continuously using an Infinite M1000 microplate reader (Tecan) at excitation and emission wavelengths of 360 and 465 nm, respectively. All measurements were performed in triplicate and results normalized to protein concentration.

### Molecular modeling

A spatial model of SmSP1 was constructed using the template X-ray structure of bovine trypsin in complex with the peptidyl inhibitor leupeptin (PDB entry 1JRT) and utilizing a pairwise sequence alignment generated by the BLAST program (BLOSUM62 substitution matrix). The homology module of the MOE program was used for modeling the SmSP1 structure (MOE: Chemical Computing Group; http://www.chemcomp.com). The conformation of leupeptin was refined by applying the LigX module of the MOE. The final binding mode of the inhibitor was selected by the best fit model based on the London dG scoring function and the generalized Born method [41]. Molecular images were generated with UCSF Chimera (http://www.cgl.ucsf.edu/chimera/). The electrostatic surface potential was calculated using the APBS software [42] and input data were prepared using PDB2PQR [43].

## Results

### Gene annotation and sequence analysis reveals complex domain organizations for some SmSPs

Genes were selected *in silico* based on a proteolytic domain organization that matched with family S1 serine proteases: cercarial elastases were excluded because of their detailed studies previously [20], [22]. The five remaining SmSP genes, including the previously sequenced and partially characterized SmSP1 [13], [14], were cloned and sequenced. The other four gene sequences named SmSP2 through SmSP5 (Table 1) were significantly corrected and re-annotated in the primary database (*S. mansoni* GeneDB) due to various sequence inaccuracies. The sequences of SmSP2 through SmSP5 were deposited into the GenBank as KF510120, KF510121, KF510122, KF939306, respectively. The sequence of SmSP1 defined here was also deposited (KF535923) because of sequence differences from the original description (CAA09691 [13]) and from the information in *S. mansoni* GeneDB (Smp_030350; Figure S1). A search of the *Schistosoma japonicum* genome [44] indicates that orthologs for each of the SmSPs are present; SjSP1 (GeneDB Sjp_0012180, GenBank N/A), SjSP2 (Sjp_0100980, CAX74751), SjSP3 (Sjp_0023390, CAX73257), SjSP4 (Sjp_0047680, N/A) and SjSP5 (Sjp_0114710, CAX73292).

**Figure 1:**
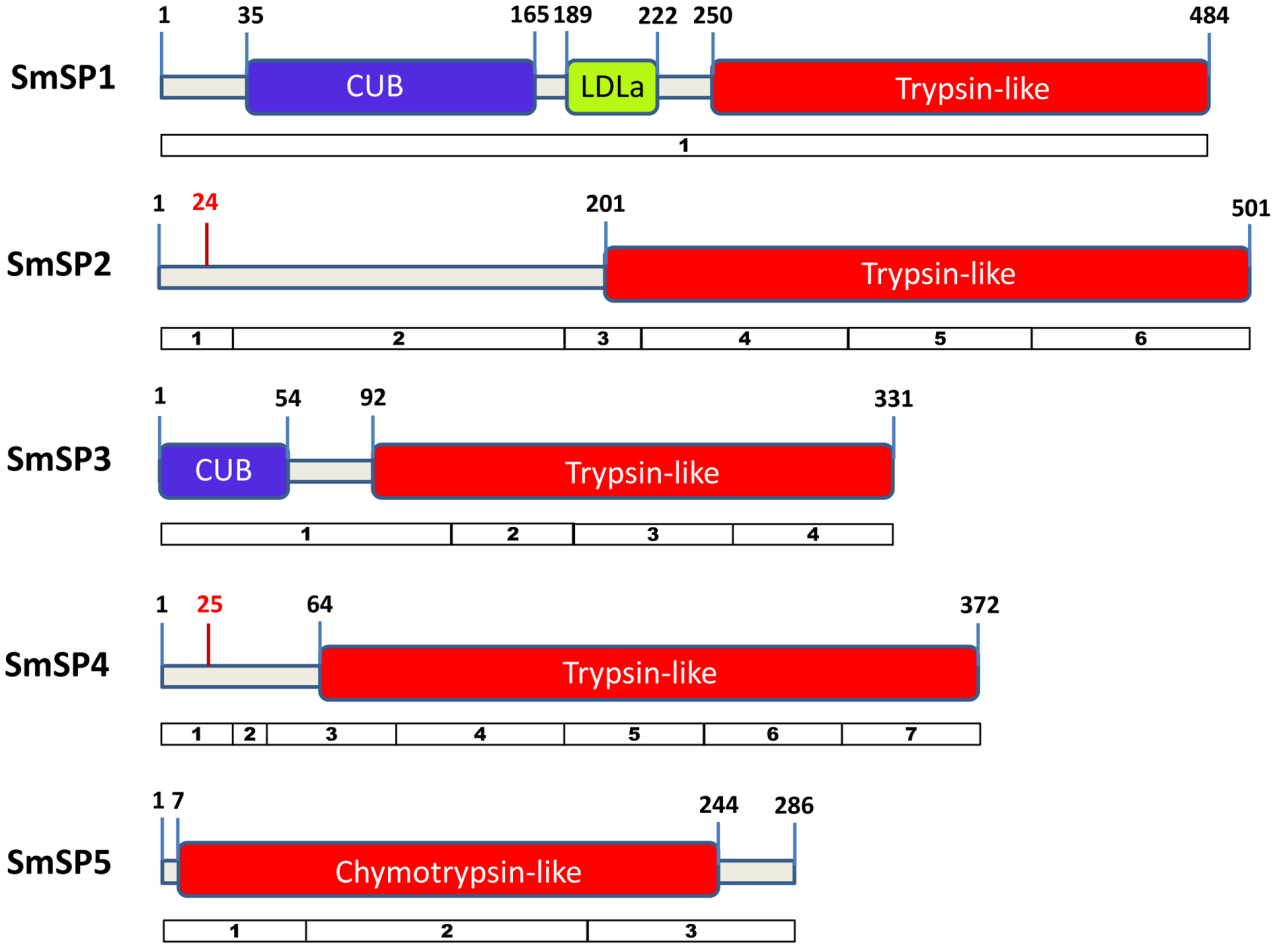
Predicted domain organization and open reading frames of SmSP proteases. CUB domains are depicted in blue, an LDLa domain in yellow and protease domains from the S1 family in red. In SmSP2 and SmSP4, N-terminal signal peptides are separated by red bars from the rest of N-terminal extensions with putative pro-peptides (protease activation peptides). Numbering indicates amino acid positions. Exon structure of the genes encoding SmSPs are shown as numbered boxes below each SmSP protein.

The sequence domain organization for the particular proteases is represented in Figure 1. Based on sequence homology analysis, we describe SmSP1 as a multi-domain protein comprising a matriptase-like structure made up of **C**omplement-**U**egf-**B**MP-1 (CUB) extracellular and plasma membrane-associated domains, a LDL-binding receptor domain class A (LDLa domain) and a S1 family serine protease domain. However, the full gene product has been detected only in the eggs, whereas in other parasite stages, the CUB and protease domains are expressed as separate spliced products, as demonstrated by PCR and sequencing (Figure S2).

**Figure 2:**
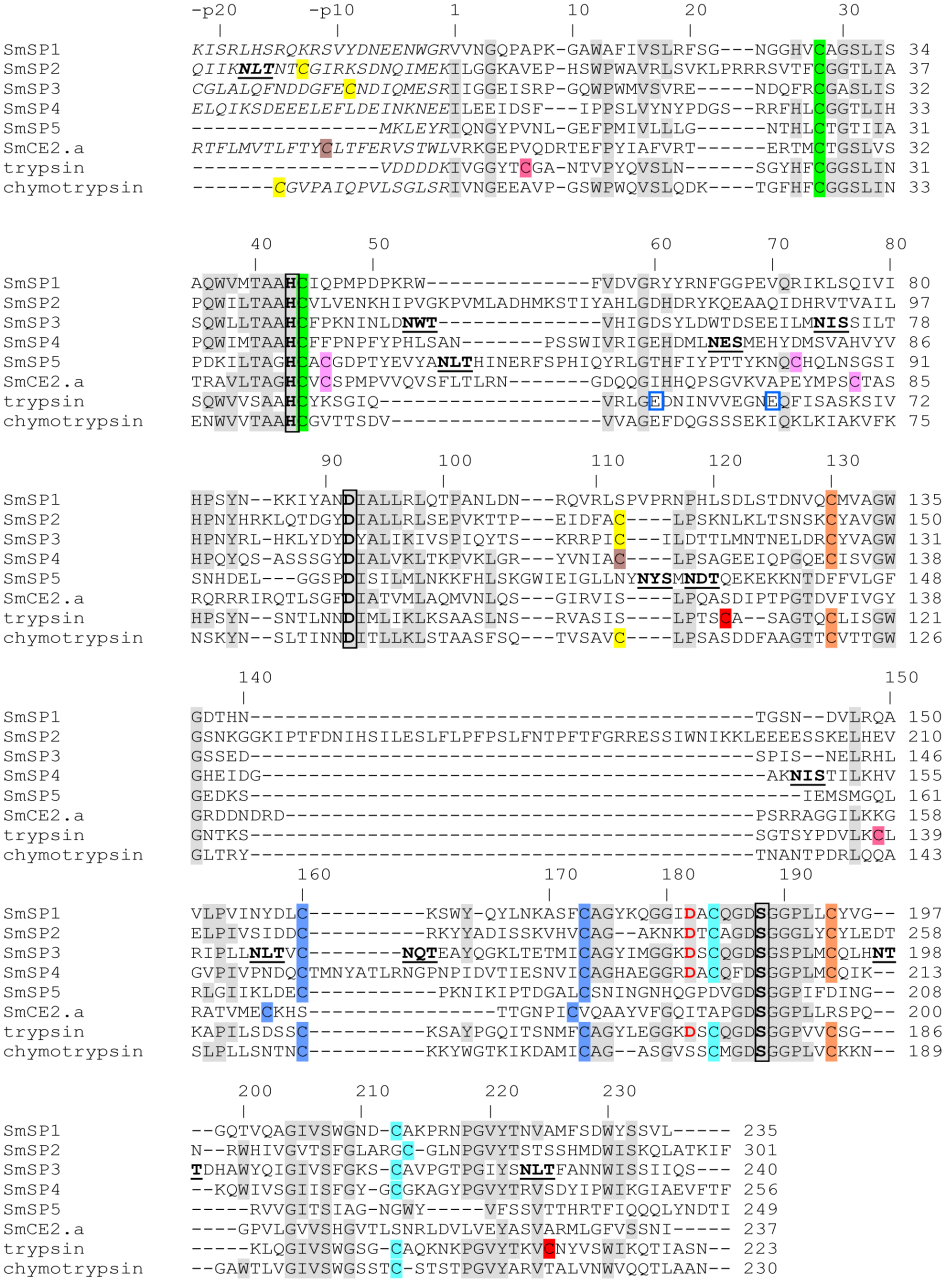
Primary sequence alignment of SmSP1 through SmSP5 with *S. mansoni* cercarial elastase 2a (SmCE2.a), bovine trypsin and bovine chymotrypsin. For SmSP1 to SmSP4, only the protease domains are shown; the upstream sequences (except a short sequence stretch) forming N-terminal extensions and non-proteolytic domains are not included in the alignment. Also, a downstream C-terminal extension of SmSP5 is not included. The catalytic residues His, Asp and Ser are highlighted in bold and black-boxed; critical Asp residues in the S1 subsite that account for trypsin-like activity are in bold red; Cys residues that are predicted to form disulfide bonds are indicated by the same color; putative unpaired Cys residues are highlighted in olive, and predicted N-glycosylation signals are in bold and underlined. Glu residues binding a Ca^2+^ ion in the trypsin molecule are blue-boxed. The upper line numbering is according to SmSP1; the predicted mature protease domain starts with 1, the suffix p indicates pro-peptide/N-terminal extension numbering. GenBank accession numbers are as follows: SmSP1 (KF535923), SmSP2 (KF510120), SmSP3 (KF510121), SmSP4 (KF510122), SmSP5 (KF939306), SmCE2a (AAM43941), bovine trypsinogen (XP_871686) and bovine chymotrypsinogen A (XP_003583409).

Primary sequence homology analysis shows that SmSP2 to SmSP5 are distinct molecules with the same family S1 type catalytic protease domain at the C-terminus, but with different N-terminal extensions which include a potential pro-peptide, i.e., a peptide that is removed during zymogen activation. The N-terminal extensions vary from 201 residues in SmSP2 to just a seven residues in SmSP5 (Figure 1). SmSP1, SmSP3 and SmSP5 do not contain a predicted signal sequence for the secretory pathway as identified by the SignalP program [45]. In contrast, SmSP2 and SmSP4 are synthesized as pre-pro-proteins with a typical N-terminal signal peptide preceding an N-terminal extension region containing a putative pro-peptide (‘activation peptide’) that is then followed by the protease domain (Figure 1). The pro-peptide is separated from the protease domain of SmSPs by a basic residue, Arg or Lys (Figure 2) which constitutes a potential activating cleavage site, i.e., is hydrolyzed during protease maturation as is known for other S1 family proteases [7]. For SmSP3, the N-terminal extension contains an incomplete CUB domain. PCR and sequencing revealed that, as found for SmSP1, the CUB and the protease domains of SmSP3 are only co-expressed in eggs whereas they are separate spliced gene products in the other stages (Figure S2). SmSP5 contains a Thr/Asn rich C-terminal sequence extension not present in orthologous SPs from other trematodes (Figure S4).

The catalytic protease domains of SmSP1 to SmSP4 share significantly greater sequence identity (about 30%) with each other than with SmSP5 (about 20%; Figure S3). All five SmSPs have a catalytic triad in the order of His, Asp and Ser that is typical for S1 family proteases; also, the regions surrounding the catalytic triad residues have the most notable sequence identity (Figure 2). The protease domains of SmSP1 to SmSP4 contain cysteine residues at positions 28, 44, 130, 160, 173, 184, 194, and 212 (SmSP1 protease domain numbering), which are conserved in other trypsin-like proteases. They form four disulfide bonds that can be predicted from the alignment with the crystal structures of bovine trypsin and bovine chymotrypsin (Figure 2). Moreover, the protease domain of SmSP2 through SmSP4 contains an additional cysteine residue, Cys112. By comparison with bovine chymotrypsin, this residue in SmSP2 and SmSP3 is likely to form a disulfide bond with a Cys in the N-terminal extension region (at the positions -p13 and -p9, respectively), whereas in SmSP4 a similar Cys in the N-terminal extension region is lacking (Figure 2).

SmSP5 diverges from the other four SPs in that it contains only six cysteine residues that likely form three disulfide bonds. The first two bonds, Cys28-Cys44 and Cys160-Cys173, are identical to those in trypsin, chymotrypsin and other SmSPs. The remaining cysteine residues (Cys46 and Cys72) are absent, but correspond to Cys46 and Cys77 in SmCE that were predicted to form a disulfide bond by homology modeling [46] (Figure 2). Moreover, both SmSP5 and SmCEs lack the disulfides Cys130-Cys194 and Cys184-Cys212, which are conserved in SmSP1 to SmSP4. Taken together, SmSP5 clearly differs in its disulfide pattern from the other investigated SmSPs. This close structural relationship between SmSP5 and the SmCEs is confirmed for the other analyses performed (see below). In addition, two other splice variants of SmSP5 were detected. Compared to the full-length SmSP5, both are C-terminally truncated and one is missing the crucial His residue from the catalytic triad (Figure S4).

Asp182 determines the trypsin-like specificity of serine proteases for substrates with Arg/Lys in the P1 position [47], and this residue is conserved in all of the SmSPs except SmSP5 (Figure 2), which has Gly. Therefore, it might be the case that SmSP5 displays a substrate specificity similar to that of chymotrypsin/elastase-type proteases which also contain a hydrophobic/uncharged residue in the position 182. The calcium binding site in mammalian trypsins is formed mainly by Glu70 and Glu80 (trypsin numbering, corresponding to Glu60 and Glu70 in SmSP1) [48]. This motif is not strictly conserved in the analyzed SmSP sequences; however, it might be present in a modified functional form in SmSP2, SmSP3 and SmSP4 that contain acidic residues in the close proximity of those locations (Figure 2).

### SmSPs, including their domains, are differentially expressed across developmental stages

**Figure 3:**
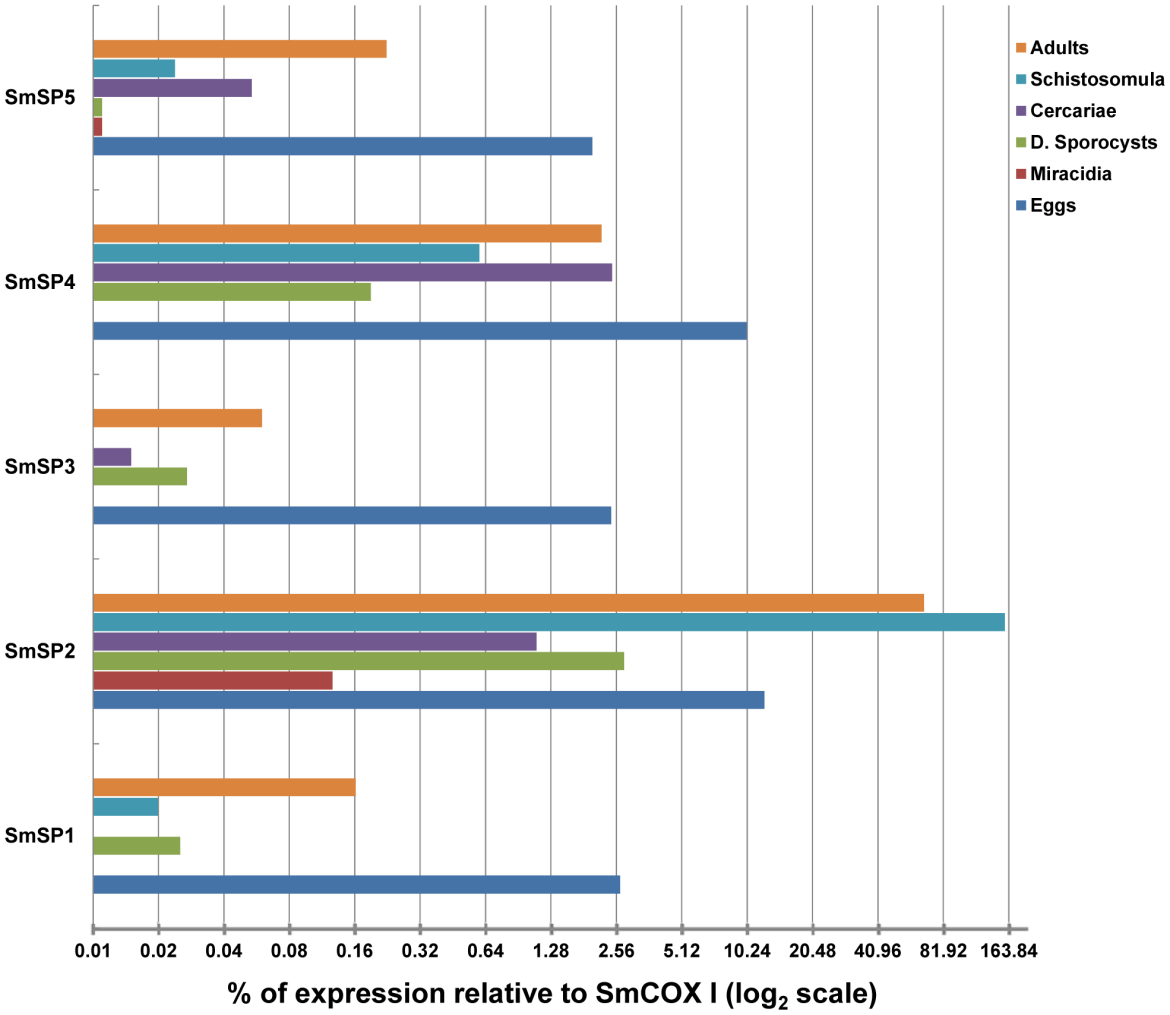
RT-qPCR to evaluate the expression of SmSP genes among *S. mansoni* developmental stages. mRNA levels are displayed as the percentage of expression compared to the constitutively expressed *S. mansoni* cytochrome oxidase I (SmCOX I). The value 0.01% was used as a significance threshold. The gene expression analysis of the protease domains of SmSPs. Each unit represents the -fold change in the transcription level using the log_2_ scale.

**Figure 4:**
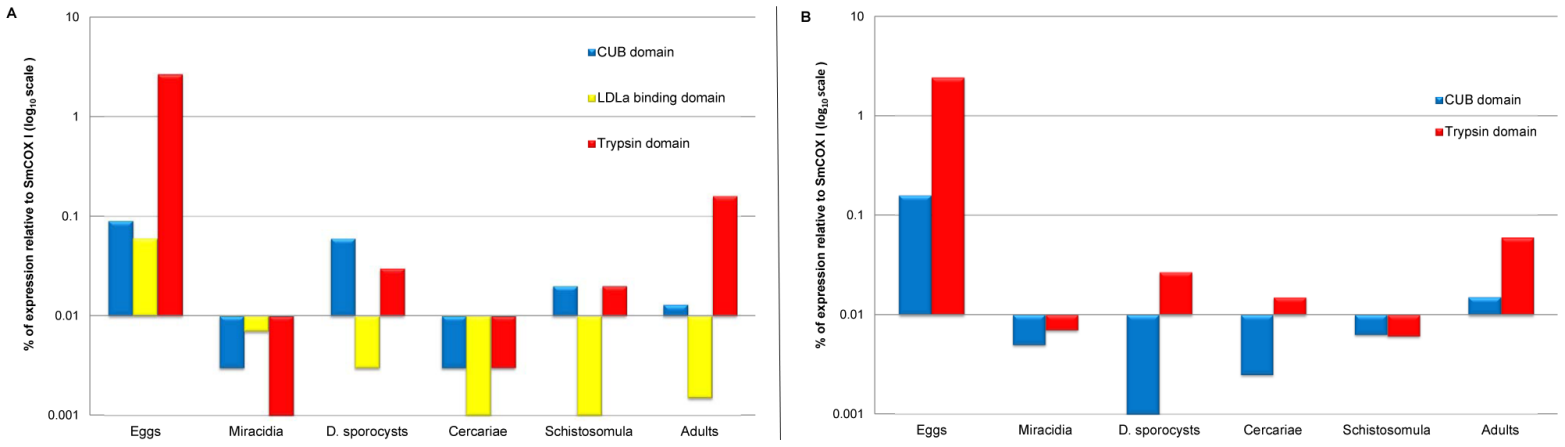
Comparisons of mRNA levels for the separate domains of SmSP1 (A) and SmSP3 (B) displayed in the log_10_ scale and as a percentage of SmCOX I expression level. D. sporocyst = daughter sporocyst.

Messenger RNA transcript levels for the five SmSPs were evaluated in eggs, miracidia, daughter sporocysts, cercariae, schistosomula and adults using RT- qPCR (Figure 3). For SmSP1 and SmSP3, we determined gene expression for both the protease and non-protease domains (Figure 4).

For SmSP1, the greatest expression was recorded in eggs at 2.5% of the expression level of the reference gene, SmCOX I. Low expression was recorded in adult worms, five-day old schistosomula and daughter sporocysts at around 0.1% or below relative to SmCOX I. Expression in the other stages was below significance, i.e., less than 0.01% of SmCOX I. As described above, the ORF of SmSP1 consists of 3 domains and their individual expression was evaluated by RT-qPCR and PCR (Figure 4A; Figure S2). The data show a differential expression pattern for the CUB, LDLa and protease domains of SmSP1: expression of the CUB domain is mostly in eggs and sporocysts, whereas LDLa is only expressed in eggs with an expression level about 20-fold lower than that of the protease domain (Figure 4A). As stated above, only in eggs is the whole ORF amplified by PCR suggesting that some SmSP1 is expressed as the full-length multi-domain protein (Figure S2).

Among the SmSPs, SmSP2 is the most abundantly expressed SmSP (Figure 3). In fact, expression in schistosomula and adults is on a similar level to that previously measured for the well-characterized *S. mansoni* cysteine and aspartic proteases [27]. In adults, SmSP2 expression is equivalent to that of SmCOX I, whereas in five-day old schistosomula expression is even greater - 150% that of SmCOX I. Significant expression, i.e., 10% that of SmCOX I, is also detected in eggs. In the other stages, expression is close to or below 1% of the SmCOX I level.

The expression pattern of SmSP3 across all life stages is similar to that of SmSP1 (Figure 3), with minor variations regarding expression in cercariae and schistosomula. Most expression is found in eggs at 2.5% of the SmCOX I expression level. Interestingly, the CUB and protease domains are only co-expressed in eggs and adults (Figure 4B), whereas differential expression is seen for the other developmental stages (Figure S2). SmSP4 is expressed predominantly in eggs (around 10% of SmCOX I level). For the other stages, approximately 1–2% of the SmCOX I level is detectable in cercariae, adults and five-day old schistosomula. Finally, SmSP5 is expressed predominantly in the eggs (2% of the level of SmCOX I) with low expression in the other life stages (0.02–0.05% of SmCOX).

### Phylogenetic position of SmSPs: SmSP5 as ‘a missing-link’ chymotrypsin-like protease

**Figure 5:**
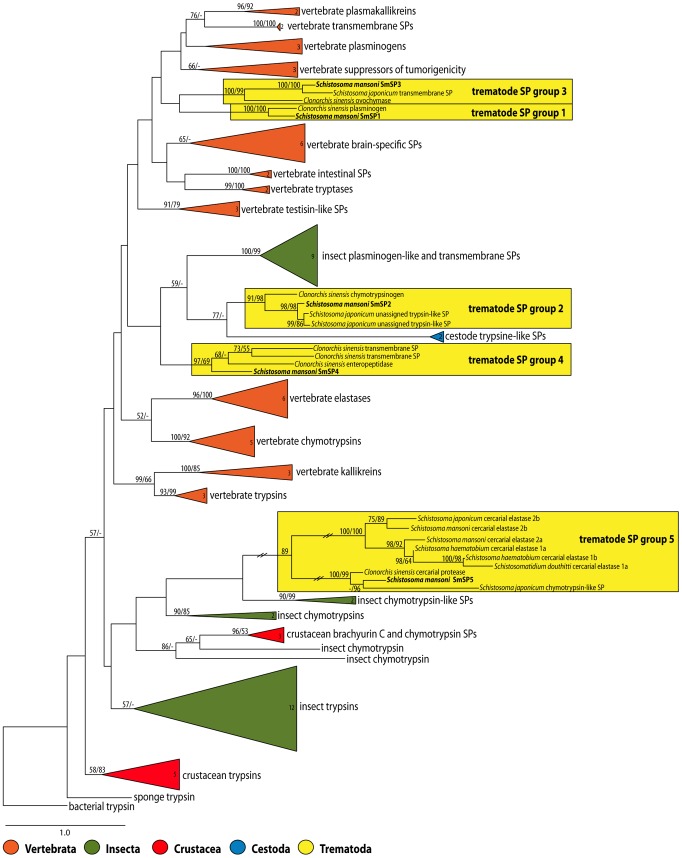
Maximum likelihood phylogenetic tree of 101 selected members of the S1 family of serine proteases with emphasis on trematode SPs. Numbers in the collapsed branches (triangles) indicate the number of taxa included in the branch. Maximum likelihood and maximum parsimony bootstrap supports are shown at nodes, bootstrap percentages with <50% support are not shown. Branches in the trematode clade 5 SPs are shortened to one third of their original length as indicated by the two diagonal lines. For clades 1 and 4, two *S. japonicum* orthologs are missing due to their absence in the GenBank nr database. However, both sequences can be retrieved from the SchistoDB database under the identifiers Sjp_0012180 (SjSP1) and Sjp_0047680 (Sj SP4).

The maximum likelihood analysis of a wide spectrum of vertebrate and invertebrate S1 family SPs based on amino acid sequences revealed that SmSPs clustered with related trematode proteases into five distinct and well-supported clades (Figure 5). Identical results were obtained using maximum parsimony analysis (data not shown). The clades did not create a monophyletic group. Thus, SmSP1 and SmSP3 were placed as two closely related but independent clades (trematode SP clade 1 and 3) and clustered with a large group of vertebrate SPs, including regulatory- and epithelial-derived effector trypsin-like proteases such as plasminogens, plasma kallikreins, tryptases, matriptases and transmembrane SPs (Figure 5). SmSP2 and SmSP4 also segregated into two separate but related trematode clades (numbers 2 and 4), which clustered with cestode SPs and a group of insect plasminogen-like and trans-membrane SPs (Figure 5). Finally, SmSP5 clustered with *S. japonicum* and *Clonorchis sinensis* (Chinese liver fluke) orthologs and created a sub-clade that grouped with a sub-clade of CEs within the trematode SP clade 5. This clade also clustered with chymotrypsin-like proteases from invertebrates. Accordingly, SmSP5 and its trematode orthologs associate more with the divergent schistosome CEs [22] than with other S1 family proteases [18].

### Activity profiling demonstrates trypsin-like proteases in *S. mansoni* developmental stages

S1 family SP activities in soluble extracts of *S. mansoni* adults, five day-old schistosomula and eggs were profiled for proteolytic specificity using peptidyl fluorogenic substrates. Two sets of specific protease substrates were used; (i) substrates with a basic amino acid residue (Arg, Lys) in the P1 position that are cleaved by trypsin-like SPs, and (ii) substrates containing bulky hydrophobic (Phe, Tyr) or aliphatic residues (Val, Leu, Met) at P1 that are cleaved by chymotrypsin- or elastase-like SPs [49]. The measured activities were further authenticated as S1 family SPs by their sensitivity to the small molecule inhibitors, Pefabloc SC and PMSF.

**Figure 6:**
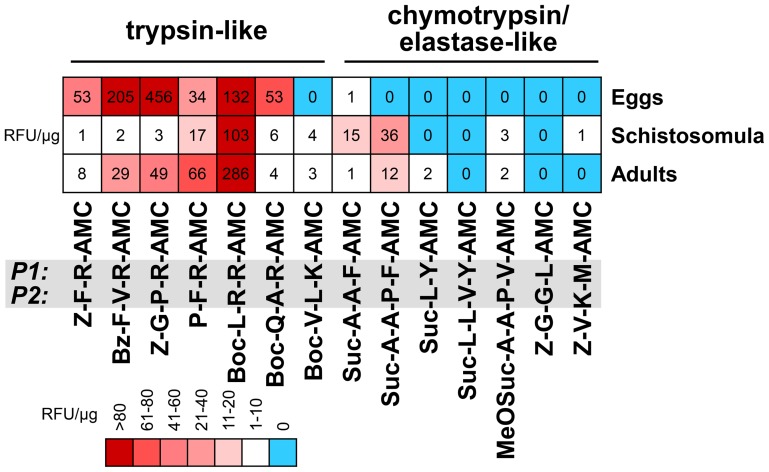
Profiling SP activities in extracts of *S. mansoni* developmental stages. The kinetic assays, performed at pH µM fluorogenic substrates (P1 and P2 positions are highlighted) that are specific for trypsin- and chymotrypsin/elastase-type proteases. SP activities (sensitive to inhibition by PMSF and Pefabloc SC) are expressed as relative fluorescence units (RFU/s) and normalized to the protein content of extracts. Data are displayed in a heat map.

The results indicate that trypsin-like activities predominate over chymotrypsin/elastase-like activities in the analyzed extracts (Figure 6). The trypsin substrates were hydrolyzed with variable efficiencies giving distinct cleavage patterns for the individual life stages. The prominent activity in all extracts was best measured with the Boc-L-R-R-AMC substrate, hence making this substrate a useful probe to detect and measure SmSPs. Extracts of eggs displayed a particularly complex profile by cleaving an additional two substrates, Bz-F-V-R-AMC, and Z-G-P-R-AMC. This suggests that this life-stage possesses additional, possibly stage-specific, trypsin-like proteases. In contrast to the major trypsin-like activities, chymotrypsin/elastase-like activity was relatively weak being measured only in schistosomula and adults.

Subsequently, we tested whether SmSPs is measurable in the E/S products from eggs, schistosomula and adults. For this purpose, we used the substrate Boc-L-R-R-AMC, which was identified as the most efficient substrate for homogenates of all the life stages (Figure 6). The specific activities of the E/S products, which were inhibited by the SP inhibitors, Pefabloc SC and PMSF, were 1.05±0.10, 1.38±0.05, and 0.11±0.01 RFU/µg protein for eggs, schistosomula and adults, respectively.

### Spatial structure modeling predicts a trypsin-like substrate specificity of SmSP1

A spatial homology model of the protease domain of SmSP1 was constructed to analyze its binding pocket and substrate specificity. The X-ray structure of bovine trypsin in complex with the small-molecule inhibitor, leupeptin (PDB code 1jrt), was used as a template. We used SmSP1 as representative of SmSP1 to SmSP4, which have substantial sequence identity, a similar disulfide pattern and homology in active site regions (Figures 2 and S3). Figure S5 shows that the SmSP1 protease domain displays the conserved architecture of S1 family proteases which consists of two six-stranded β-barrel domains packed against each other. The catalytic amino acid residues are located at the junction between the domains. The major insertion/deletion variations between SmSP1 to SmSP4 (such as the SmSP2 insertion at residue 140, Figure 2) are located at surface-exposed loops.

**Figure 7:**
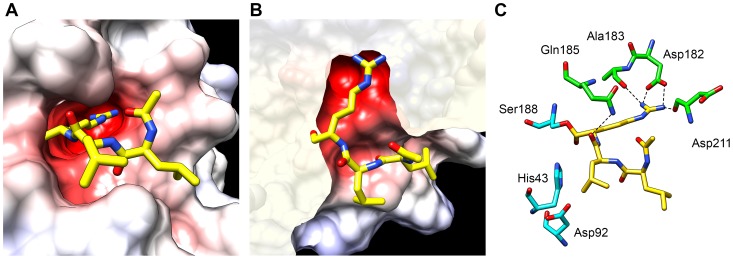
Homology model of the SmSP1 protease domain in complex with leupeptin. The model was built using the template X-ray structure of bovine trypsin in complex with substrate-like inhibitor leupeptin (N-acetyl-L-leucyl-L-leucyl-L-argininal; PDB code 1jrt). (**A**) Surface representation of the SmSP1 active site colored by electrostatic potential (at a scale from −10 kT/e (red) to +10 kT/e (blue)). Carbon atoms of leupeptin are yellow; heteroatoms have a standard color coding (O, red; N, blue). (**B**) The same detail as (A) but viewed from above (the surface display was clipped for a better view). (**C**) Schematic view of the active site residues of SmSP1 (green) forming hydrogen bonds (dashed lines) with leupeptin (yellow). Note the interactions between Asp182 (in the S1 protease subsite) and the basic P1 residue of leupeptin that mimic the S1-P1 salt bridge that is critical for trypsin-like substrate specificity. Catalytic residues (cyan) are shown, including the covalent linkage of leupeptin with the catalytic Ser188.

The primary substrate specificity determinant of S1 family proteases is the S1 binding subsite. In SmSP1, this subsite forms a deep and narrow negatively charged pocket that contains Asp182 at the bottom (Figures 7A and 7B). Leupeptin, the transition state analog protease inhibitor, was docked into the active site of SmSP1. The arginal residue of leupeptin forms a covalent linkage with the catalytic Ser188, a salt bridge with Asp182 in the S1 subsite and hydrogen bonds with the carbonyl oxygen of Ala183 and Asp211 (Figure 7C). An additional hydrogen bond is formed between the side chain nitrogen of Gln185 and the carbonyl oxygen Leu2 residue of leupeptin. The putative interaction pattern of leupeptin at the S1 subsite of SmSP1 is similar to that found in bovine trypsin [50]. This demonstrates that SmSP1 has a substrate binding preference for basic residues at the P1 position, the positive charge of which compliments the negatively charged Asp182, i.e., trypsin-like activity. This conclusion can be generalized to SmSP2 to SmSP4 which also contain the critical Asp182 residue.

## Discussion

Much has been reported on the genetic, biochemical and functional characterization of cysteine and aspartic protease activities in schistosomes [16], [17] and flatworms in general [16], [51], and of the schistosome CE SPs [20] that putatively facilitate parasite invasion of the mammalian host [18]–[20]. By comparison, relatively little detail is available for non-CE SPs. There are, however, indications that non-CE S1 family SPs contribute to successful infection [6]. Thus, kallikrein-like protease activity from *S. mansoni* adults [12] and plasmin-like fibrinolytic activity from *S. mansoni* eggs [15] have been recorded previously. Both activities displayed trypsin type cleavage specificities and both may contribute to the phenomenon, whereby large occlusions of veins by schistosomes are not associated with intravascular deposition of fibrin and thrombus formation [52]–[54]. At the gene and primary sequence levels, however, only two SmSPs, namely SmSP1 [13], [14] and another [23], [24], which we term SmSP2, have been described.

The *S. mansoni* GeneDB currently contains 16 unique sequences that belong to Clan PA family S1 SPs. This number is significantly lower than the 135 family S1 proteases found in the human genome [8], [25] and may be due to the lack of need to regulate the more complex and expanded physiological processes found in vertebrates [55]. In our study and apart from SmSP1 [13], [14], we identified four additional SmSP genes encoding typical sequence features of the S1 family [7], [8] and which we term SmSP2 through SmSP5. Two further genes (Smp_194090 and Smp_06530 in GeneDB) were identified in the *S. mansoni* GeneDB as putative proteolytically inactive SmSPs as they lack the catalytic serine or histidine residue in the catalytic triad. The remaining nine of the 16 family S1 SPs comprise eight CEs (encoding both putative proteolytically active and inactive products) and a gene (Smp_174530) that encodes an S1 family SP ORF fused downstream of an M01 family metallo-protease. This protease that was not known to us at the beginning of our study and because of its domain complexity and sequence size was not described further.

Our phylogenetic analyses of trematode SPs displayed interesting evolutionary trends. The SmSPs segregate into five clusters of family S1 proteases. The protease domains of SmSP1 and SmSP3, forming clades 1 and 3, respectively, cluster with a large group of vertebrate trypsin-like SPs including regulatory and effector epithelial-derived proteases. In addition to a protease domain, the ORFs for SmSP1 and SmSP3 include non-catalytic CUB domains and SmSP1 LDLa domain. Several vertebrate matriptases that also contain CUB domains are present in our phylogenetic analysis including those belonging to the ‘suppressor of tumorigenicity’ group. As judged by the domain organization, SmSP1 resembles mammalian matriptases (a.k.a. epithin, MT-SP); however unlike conventional matriptases with multiple CUB and LDLa domains, SmSP1 has only one of each. CUB domains were first described in the complement proteins C1r and C1s and are modules of approximately 110 amino acids with four conserved cysteine residues [56]. These domains mediate protein-protein interactions and are generally associated with proteins that have diverse, usually regulatory, functions in the extracellular space and/or plasma membrane [56]. CUB domains can also interact with heparin and glycoproteins [56] and are often associated with metallo-proteases, in addition to serine proteases [8].

Based on the RT-qPCR analysis, the complete ORFs of SmSP1 and SmSP3 molecules share a similar expression profile (quantitatively and, to a smaller degree, qualitatively) across the developmental stages tested. However, it is also clear that the individual protease, CUB and/or LDLa domains are differentially expressed across the developmental stages tested being only co-expressed in eggs and, for SmSP3, adults. The particular functions of these enzymes and their component domains are unknown and their importance to parasite vitality and/or survival might be tested via specific RNA interference (RNAi), which has been shown to operate in schistosomes [30], [57], [58]. According to our phylogenetic analysis, the closest vertebrate orthologs to SmSP1 and SmSP3 are those associated with regulatory cascades such as fibrinolysis and vasodilation. This, together with the fact that SmSP1 was detected apparently on the surface area of worms and secreted into the cultivation media [13], suggests a possible function at the host-parasite interface.

The presence in the ORF of SmSP1 of an LDLa domain (positioned between the CUB and catalytic domains) deserves a note. Schistosomes and other flatworms do not synthesize cholesterol (found within LDL) and must therefore scavenge it from the environment, particularly for the energy-intensive work of producing eggs [59], [60]. There is also a report that the presence of *S. mansoni* eggs is connected with decreased circulating levels of cholesterol in the host [61], however, we can only speculate about the real function of the SmSP1 LDLa domain.

SmSP2 and SmSP4 form two other separate clades and cluster with trypsin SPs from insect and other invertebrates. Both proteases are characterized by their longer but different N-terminal extensions that lack homologies to known proteins but which are shared in orthologous SPs from *S. japonicum* [44] and *C. sinensis* [62]. Functions as yet are unknown, however, it is certainly remarkable that SmSP2 is massively expressed in schistosomula and adults (150% and 60% of SmCOX I expression levels, respectively) and, therefore, conceivably contributes significantly to host and/or parasite protein hydrolysis, perhaps in modulating of host physiologic processes [6], [12]. The presence also of close orthologs of SmSP2 in *Fasciola gigantica* [63] and *C. sinensis* [62] suggests a general role for SP2 during infection in the mammalian host. The impressive expression levels for SmSP2 are consistent with high levels of SmSP2 expression from microarray [23] and transcriptome data [24]. Also, the expression levels are close to those for the gut-associated, digestive cysteine and aspartic proteases, SmCB1 and SmCD, respectively, for which expression is close to that of SmCOX I [27].

Finally, for SmSP5, phylogenetic analysis identified its relative position in what we term clade number 5. This clade is most closely related to chymotrypsins from invertebrates and comprises SP5 orthologs in *S. japonicum* [44] and *C. sinensis* [62], and the CE genes in *S. mansoni, S. haematobium* [20], [22], *S. japonicum* [44] and *Schistosomatium douthitti* [20]. Clade 5 is particularly significant for phylogenetic relationship studies of schistosome proteolytic enzymes as it contains sequences that bridge the outlier CE group and other ‘more typical’ S1 family SPs. Specifically, our previous phylogenetic work [18] had highlighted that the CEs coalesce as a tight group that diverges from other family S1 protease sequences. At that time the SmSP5 sequence was incomplete and not amenable to analysis [18]. The current sequence analysis suggests that SmSP5 and its trematode orthologs are ‘a missing link’ between the outlier CE group and the common ancestor. CEs initially evolved from chymotrypsin regulatory proteases and may provide an evolutionary advantage in contributing to host invasion [22].

For the SmSP protease domains, we investigated the structure-function relationships using primary structure analysis, homology modeling and protease activity profiling with peptidyl substrates. The sequence alignment shows that all the SmSPs except SmSP5 share a conserved Asp182 residue. This residue defines the specificity for the S1 binding site and drives a strong preference for Arg and Lys residues at the P1 position of protein/peptide substrates, as demonstrated for vertebrate trypsins [47]. The homology model of SmSP1 reveals that the S1 pocket with its critical Asp182 residue has an architecture analogous to vertebrate trypsins. In contrast, the S1 binding pocket of SmSP5 has a Gly182. Also, SmSP5 lacks the disulfide Cys184-Cys212 which is present in the other four SmSPs and known to stabilize the S1 binding site in vertebrate trypsins. Interestingly, this disulfide is also absent in schistosome CEs, which contain non-polar residues (Ile or Leu) at the bottom of the S1 binding pocket resulting in elastase and chymotrypsin-like activities [22].

Consistent with the number of trypsin-like sequences in all of the life-stages studied, major trypsin-like activities could also be measured using peptidyl fluorogenic substrates in eggs, schistosomula and adult extracts. Eggs, in particular, presented the most diverse and active profile compared to adults and schistosomula suggesting they express more than one highly active SP. Schistosomula, in contrast, displayed an activity profile restricted to one substrate, and one might suppose that this activity is in fact due to SmSP2 which was, expressed at higher levels than other SPs as measured by RT-qPCR (see above). Finally, the finding that significant trypsin-like activity was found in the E/S products of the three life stages tested suggests that one or more SmSPs are secreted into the host environment where they may interfere with relevant proteolytic cascades such as blood coagulation, complement or blood pressure regulation [6], [12].

In contrast to the trypsin-like activities measured, chymotrypsin/elastase-like activities were absent in eggs, and in schistosomula were at least one order of magnitude weaker. It is possible that the activity in schistosomula is, in whole or part, due to residual CE activity carried forward after mechanical transformation of cercariae and *in vitro* culture of schistosomula. In adults, however, this possibility seems remote and the minor activities measured may be contributed to by SP5.

To conclude, the present study provides a comprehensive phylogenetic, transcriptomic and functional framework illustrating the heretofore unknown complexity of schistosome S1 family SPs, other than the well-studied CEs [20], [22]. The individual enzymes underlying the activities measured remain ‘undiscovered country’ both in terms of their functional characterization and, not least, their possible contributions to successful parasitism by the schistosome, including at the host-parasite interface.

## Supporting Information

Figure S1 **Amino acid sequence alignment of three versions of SmSP1.** Original description (CAA09691), the *S. mansoni* GeneDB (SchistoDB, Smp_030350) description (both Puerto Rican isolates) and our current version (KF535923) sequenced from a Liberian *S. mansoni* isolate. Sequence variations are highlighted in green, turquoise and grey for KF535923, Smp_030350 and CAA0969, respectively. The CUB, LDLa and trypsin domains are underlined in blue, yellow and red, respectively.
(TIF)

Figure S2 **Expression of SmSP1 (CUB, LDLa and protease domains) and SmSP3 (CUB and protease domains) using PCR.** Primers were designed to amplify particular domains, partial or whole ORF fragments from cDNA of various *S. mansoni* developmental stages. The lanes are as follows: 1, SmSP1CUB; 2, SmSP1CUB-LDLa; 3, SmSP1trypsin; 4, SmSP1trypsin-LDLa, 5, whole ORF SmSP1CUB-LDLa-trypsin; 6, SmSP3CUB; 7, SmSP3 trypsin and 8, whole ORF SmSP3 CUB-trypsin.
(TIF)

Figure S3 **Matrix of amino acid sequence identities used in** **Figure 2** **.**
(TIF)

Figure S4 **Amino acid sequence alignment of three splice versions of SmSP5.** The catalytic residues His, Asp and Ser are highlighted.
(TIF)

Figure S5 **Comparison of the structures of SmSP1 and bovine trypsin.** A stereo image displaying a superimposition of Cα traces of the homology model of SmSP1 (cyan) and the crystal structure of trypsin (PDB code 1JRT; magenta). The catalytic residues are shown as ball and sticks (SmSP1 in green, trypsin in yellow). Disulfide bonds are depicted in blue (SmSP1) and orange (trypsin).
(TIF)

Table S1 **List of primers used for RT-qPCR analysis.**
(PDF)

Table S2 **The list of family S1 proteases (SPs sequences) used for the phylogenetic analysis.**
(PDF)
