# Supplementary figures and images for: Trypsin- and Chymotrypsin-Like Serine Proteases in Schistosoma mansoni – ‘The Undiscovered Country’
Source: PLoS Negl Trop Dis. 2014 Mar 27;8(3):e2766. doi: 10.1371/journal.pntd.0002766 (PMC3967958; doi:10.1371/journal.pntd.0002766)

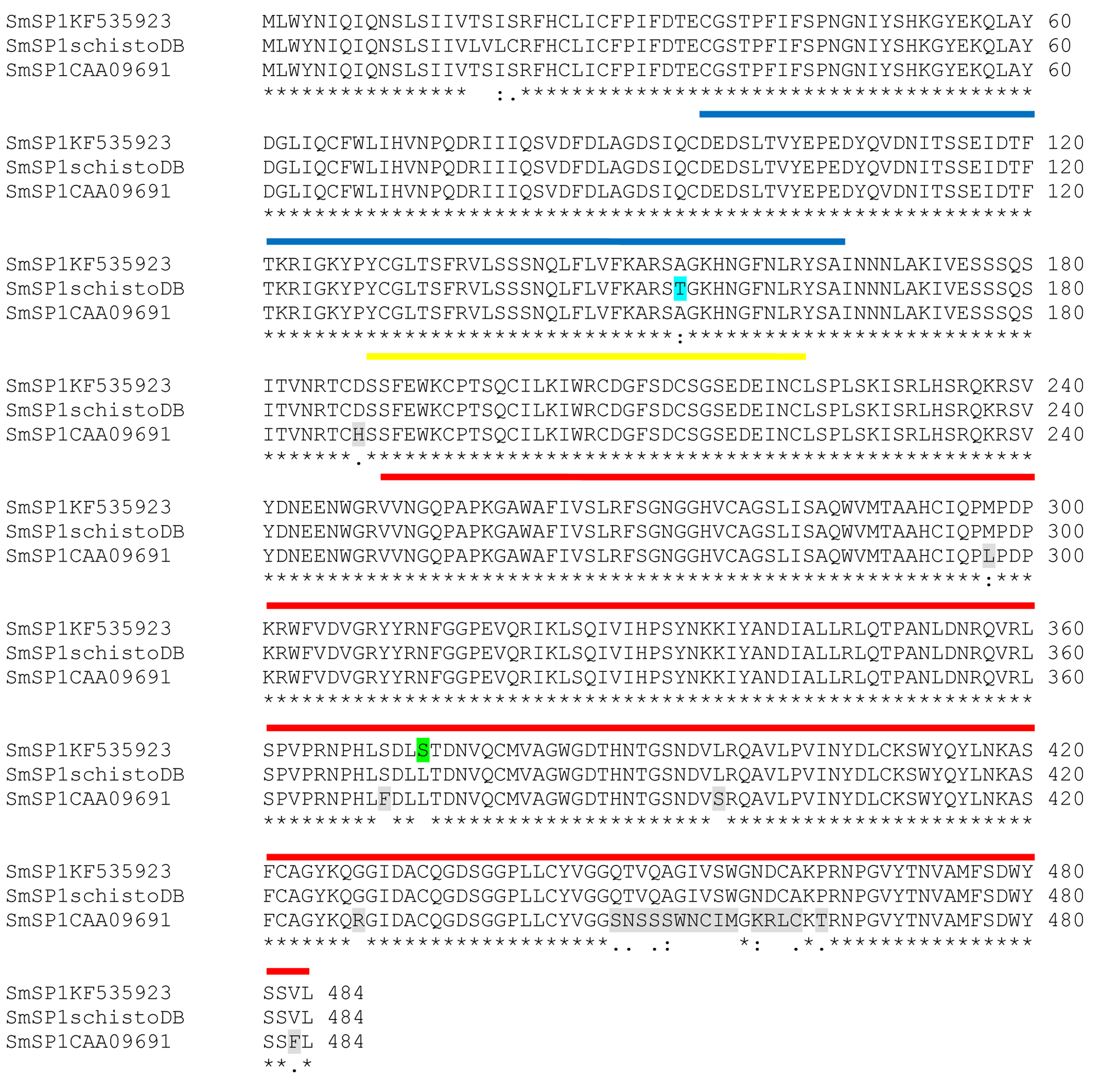

Supplement: Figure S1 — Amino acid sequence alignment of three versions of SmSP1. Original description (CAA09691), the S. mansoni GeneDB (SchistoDB, Smp_030350) description (both Puerto Rican isolates) and our current version (KF535923) sequenced from a Liberian S. mansoni isolate. Sequence variations are highlighted in green, turquoise and grey for KF535923, Smp_030350 and CAA0969, respectively. The CUB, LDLa and trypsin domains are underlined in blue, yellow and red, respectively. (TIF) [file pntd.0002766.s001.tif]

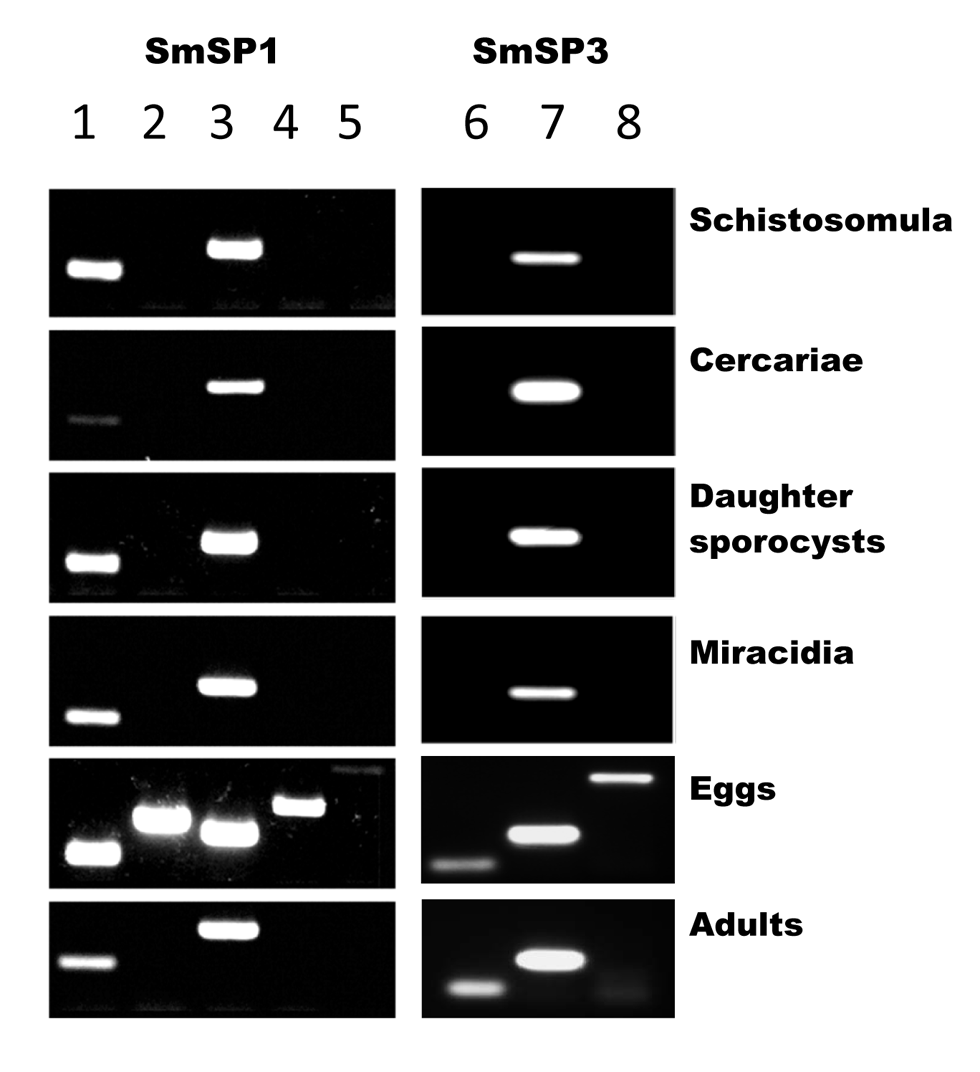

Supplement: Figure S2 — Expression of SmSP1 (CUB, LDLa and protease domains) and SmSP3 (CUB and protease domains) using PCR. Primers were designed to amplify particular domains, partial or whole ORF fragments from cDNA of various S. mansoni developmental stages. The lanes are as follows: 1, SmSP1CUB; 2, SmSP1CUB-LDLa; 3, SmSP1trypsin; 4, SmSP1trypsin-LDLa, 5, whole ORF SmSP1CUB-LDLa-trypsin; 6, SmSP3CUB; 7, SmSP3 trypsin and 8, whole ORF SmSP3 CUB-trypsin. (TIF) [file pntd.0002766.s002.tif]

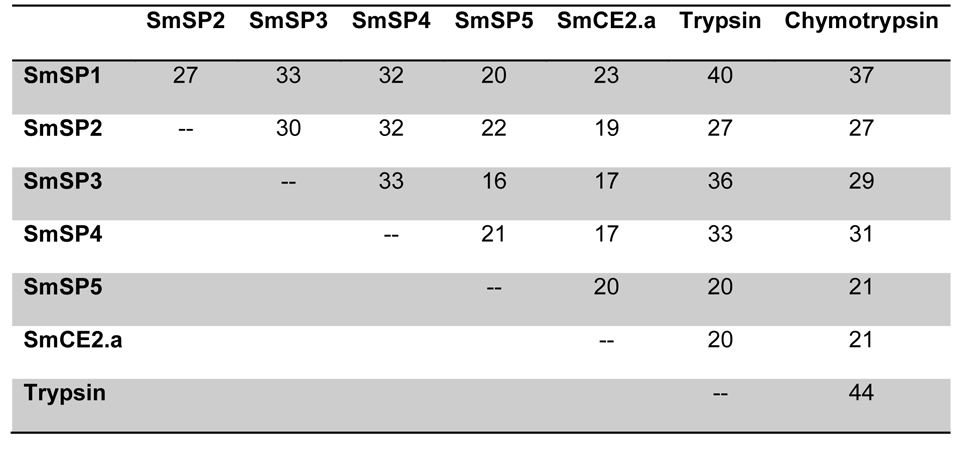

Supplement: Figure S3 — Matrix of amino acid sequence identities used in Figure 2 . (TIF) [file pntd.0002766.s003.tif]

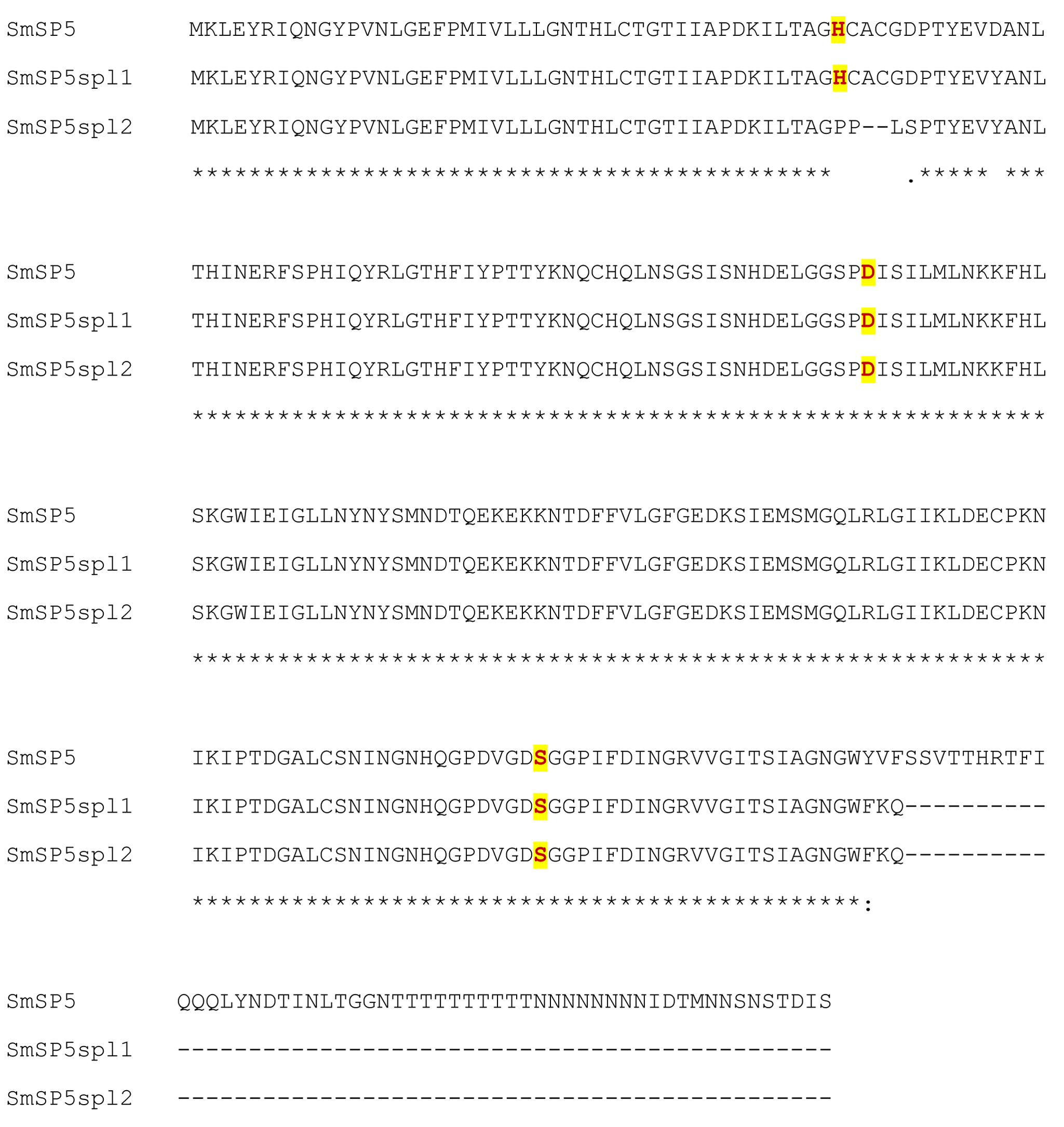

Supplement: Figure S4 — Amino acid sequence alignment of three splice versions of SmSP5. The catalytic residues His, Asp and Ser are highlighted. (TIF) [file pntd.0002766.s004.tif]

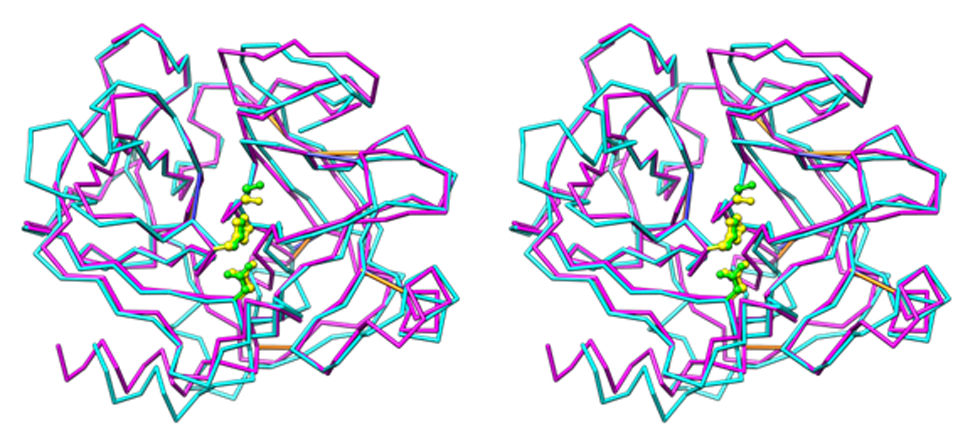

Supplement: Figure S5 — Comparison of the structures of SmSP1 and bovine trypsin. A stereo image displaying a superimposition of Cα traces of the homology model of SmSP1 (cyan) and the crystal structure of trypsin (PDB code 1JRT; magenta). The catalytic residues are shown as ball and sticks (SmSP1 in green, trypsin in yellow). Disulfide bonds are depicted in blue (SmSP1) and orange (trypsin). (TIF) [file pntd.0002766.s005.tif]
