# Supplementary material for: Trypsin- and Chymotrypsin-Like Serine Proteases in Schistosoma mansoni – ‘The Undiscovered Country’
Source: PLoS Negl Trop Dis. 2014 Mar 27;8(3):e2766. doi: 10.1371/journal.pntd.0002766 (PMC3967958; doi:10.1371/journal.pntd.0002766)
Supplement: Table S1 — List of primers used for RT-qPCR analysis. (PDF) [file pntd.0002766.s006.pdf]

Primer set	Forward qPCR primers	Reverse qPCR primers
**SmSP1cub**	TTTCCAATCTTTGATACTGAATGTG	ACGATCTTGTGGATTAACATGAATTA
**SmSP1ldla**	TCGAATGGAAATGTCCAACA	GCCCCAGTTTTCTTCGTTATC
**SmSP1trypsin**	TGGGGAGATACACATAATACAGG	GCCCCATGATACAATTCCAG
**SmSP2**	GCTGCACAAATTGATCATCG	ACCCCAACCAACAGCATAAC
**SmSP3cub**	TGGTCAATTAAAATTACCTAATCCAA	GAAGAAGAAAACCTTTTCCATGA
**SmSP3trypsin**	GGACAGTCCACATTGGTGATT	CCCATCCTGCAACATAACAA
**SmSP4**	CATGTGCCACAGACATATCG	TCCACAATGGATAATGACAGC
**SmSP5**	TGTCAATGGGTCAATTACGTTT	CCAACCATTTCCAGCTATTGA
