# Supplementary material for: Trypsin- and Chymotrypsin-Like Serine Proteases in Schistosoma mansoni – ‘The Undiscovered Country’
Source: PLoS Negl Trop Dis. 2014 Mar 27;8(3):e2766. doi: 10.1371/journal.pntd.0002766 (PMC3967958; doi:10.1371/journal.pntd.0002766)
Supplement: Table S2 — The list of family S1 proteases (SPs sequences) used for the phylogenetic analysis. (PDF) [file pntd.0002766.s007.pdf]

**Group of serine proteases**	**Taxon**
**Vertebrate plasmakallikreins**	
AAA63393	*Mus musculus*
NP_001086768	*Xenopus laevis*
**Vertebrate transmembrane SPs**	
EDM10970	*Rattus norvegicus*
AAF97867	*Mus musculus*
**Vertebrate plasminogens**	
XP_003503110	*Cricetulus griseus*
BAG60586	*Homo sapiens*
CAF97472	*Tetraodon nigroviridis*
**Vertebrate suppressors of tumorigenicity**	
XP_416635	*Gallus gallus*
XP_003459181	*Oreochromis niloticus*
XP_003222437	*Anolis carolinensis*
**Trematode SP group 3**	
KF510121	*Schistosoma mansoni*
CAX73257	*Schistosoma japonicum*
GAA27232	*Clonorchis sinensis*
**Trematode SP group 1**	
GAA38267	*Clonorchis sinensis*
KF535923	*Schistosoma mansoni*
**Vertebrate brain-specific SPs**	
XP_510756	*Pan troglodytes*
XP_001498424	*Equus caballus*
NP_001100454	*Rattus norvegicus*
XP_003478171	*Cavia porcellus*
XP_001371793	*Monodelphis domestica*
CAJ83405	*Xenopus (Silurana) tropicalis*
**Vertebrate intestinal SPs**	
CAB56465	*Mus musculus*
XP_003509591	*Cricetulus griseus*
**Vertebrate tryptases**	
2BM2_A	*Homo sapiens*
NP_999356	*Sus scrofa*
**Vertebrate testisin-like SPs**	
XP_003438688	*Oreochromis niloticus*
AAI33113	*Danio rerio*
ACO14162	*Esox lucius*
**Insect plasminogen-like and transmembrane SPs**	
XP_003398242	*Bombus terrestris*
XP_003485973	*Bombus impatiens*
XP_393727	*Apis mellifera*
EFN72139	*Camponotus floridanus*
EGI71013	*Acromyrmex echinatior*
XP_001661604	*Aedes aegypti*
XP_001847507	*Culex quinquefasciatus*
XP_002021180	*Drosophila persimilis*

EFR21103	*Anopheles darlingi*
**Trematode SP group 2**	
CAX74751	*Schistosoma japonicum*
AAW24683	*Schistosoma japonicum*
KF510120	*Schistosoma mansoni*
GAA32831	*Clonorchis sinensis*
**Cestode trypsine-like SP**	
ADP89566	*Taenia solium*
AAL14214	*Echinococcus granulosus*
**Trematode SP group 4**	
GAA56573	*Clonorchis sinensis*
GAA50807	*Clonorchis sinensis*
GAA50619	*Clonorchis sinensis*
KF510122	*Schistosoma mansoni*
**Vertebrate elastases**	
AAT45251	*Sparus aurata*
P00773	*Rattus norvegicus*
XP_002716042	*Oryctolagus cuniculus*
P08861	*Homo sapiens*
P08419	*Sus scrofa*
P00774	*Rattus norvegicus*
**Vertebrate chymotrypsins**	
P17538	*Homo sapiens*
P00767	*Bos taurus*
P47796	*Gadus morhua*
XP_511044	*Pan troglodytes*
XP_003225400	*Anolis carolinensis*
**Vertebrate kallikreins**	
P00759	*Homo sapiens*
P06870	*Homo sapiens*
P49862	*Homo sapiens*
**Vertebrate trypsins**	
P00760	*Bos taurus*
P00762	*Rattus norvegicus*
P35031	*Salmo salar*
**Trematode SP group 5**	
ACR27083	*Schistosoma japonicum*
AAM43942	*Schistosoma mansoni*
AF510342	*Schistosoma haematobium*
AAM43945	*Schistosomatium douthitti*
AAM43943	*Schistosoma haematobium*
AAM43941	*Schistosoma mansoni*
KF939306	*Schistosoma mansoni*
GAA38503	*Schistosoma japonicum*
CAX73292	*Clonorchis sinensis*
**Insect chymotrypsin-like SPs**	
AAF91345	*Glossina morsitans morsitans*
XP_002052080	*Drosophila virilis*
**Insect chymotrypsins**	

Q27289	*Anopheles gambiae*
P00769	*Vespa crabro*
**Crustacean brachyurin C and chymotrypsin SPs**	
Q8WR11	*Paralithodes camtschaticus*
P00771	*Uca pugilator*
Q00871	*Litopenaeus vannamei*
**Insect chymotrypsins**	
Q9VRT1	*Drosophila melanogaster*
XP_002046524	*Drosophila virilis*
**Insect trypsins**	
XP_001663898	*Aedes aegypti*
P29787	*Aedes aegypti*
P35040	*Anopheles gambiae*
AAV84270	*Culicoides sonorensis*
ACR61326	*Drosophila navajoa*
ABR88248	*Heliothis virescens*
AEI58562	*Eupolyphaga sinensis*
XP_003402915	*Bombus terrestris*
Q9XY56	*Ctenocephalides felis*
ACY24329	*Ctenocephalides felis*
EEZ99377	*Tribolium castaneum*
Q9XYX9	*Rhyzopertha dominica*
**Crustacean trypsins**	
Q9TY16	*Litopenaeus vannamei*
O62562	*Litopenaeus vannamei*
Q8WR10	*Paralithodes camtschaticus*
P00765	*Astacus astacus*
AAP55756	*Lepeophtheirus salmonis*
**Sponge trypsin**	
Q8I9P2	*Aplysina fistularis*
**Bacterial trypsin**	
P00775	*Streptomyces griseus*
